# *In vitro* and *in vivo* differentiation of induced pluripotent stem cells generated from urine-derived cells into cardiomyocytes

**DOI:** 10.1242/bio.029157

**Published:** 2017-12-06

**Authors:** Yu-Feng Jiang, Min Chen, Nan-Nan Zhang, Hua-Jia Yang, Qing Rui, Ya-Feng Zhou

**Affiliations:** Department of Cardiology, the First Affiliated Hospital of Soochow University, Suzhou City, Jiangsu Province, 215006, P. R. China

**Keywords:** Regenerative medicine, Noninvasive, Stem therapy, Reprogramming

## Abstract

Generation of human cardiomyocytes from cells derived from various sources, including skin biopsy, has been made possible by breakthrough advances in stem cell research. However, it is attractive to build up a negligibly invasive way to create induced pluripotent stem (iPS) cells. In this study, we created iPS cells from human urine-derived epithelial cells by gene transduction using lentiviral vectors in a totally noninvasive manner. Then, we induced the differentiation of iPS cells into functional cardiomyocytes both *in vitro* and *in vivo*. Action potentials were recorded in putative cardiomyocytes and spontaneous beating cells were observed. Our results offered an alternative method to generate cardiomyocytes in a totally noninvasive manner from an easily accessible source. The availability of urine and its potent reprogramming characteristics will provide opportunities for the use of cells with specific genotypes to study the pathogenesis and molecular mechanisms of disease *in vitro*.

## Abstract (precis)

**Summary:** We generated and verified iPS cells from urine-derived cells in a totally noninvasive manner and induced the differentiation of iPS cells into functional cardiomyocytes both *in vitro* and *in vivo*.

## INTRODUCTION

Heart failure is usually caused by loss of working myocardium and occupies high prevalence of death and disability (Roger, 2017). This is due to disability of proliferation of postnatal cardiomyocytes as they are terminally differentiated; therefore, dysfunctional or impaired cardiomyocytes cannot be self-renewed (Ellison et al., 2013).

Induced pluripotent stem (iPS) cells are biologically engineered cell types reprogrammed from somatic cells. They were first described in mice (Takahashi and Yamanaka, 2006), and soon after in humans (Yu et al., 2007; Takahashi et al., 2007). iPS cells provide an unparalleled source of patient-specific pluripotent stem cells, which is expected to advance the next generation of diagnosis and clinical regenerative medicine (Inoue et al., 2014; Nelson and Terzic, 2011). Human iPS cells have been produced from a variety of materials, including skin, embryonic tissue and umbilical cord blood (Yu et al., 2007; Takahashi et al., 2008; Aasen et al., 2008; Giorgetti et al., 2009; Haase et al., 2009; Esteban et al., 2010; Cai et al., 2010). This encourages people to assume that iPS cells can be derived from all cell types, and to find a preferred source for reprogramming. Ideal cell sources are considered to be accessible, susceptible and universal. Recently, the most common cell type for reprogramming may be dermal fibroblasts, which still requires biopsy, directly discouraging voluntary donations to organizations and research.

The human urinary system consists of a complicated small tube network, which has a larger total surface than the skin. In normal physiological conditions, thousands of cells fall down from the tubular system, ureter, bladder and urethra, and scatter daily in the urine (Rahmoune et al., 2005). Sutherland et al. first reported successful cell shedding, and multiple groups have reproduced this (Sutherland, 1972; Linder, 1976; Herz, 1980; Alberto et al., 2013; Dörrenhaus et al., 2000). It is noteworthy that urine production is an essential physiological process at any age, despite gender or ethnicity. Thus, we hypothesized that reprogramming of iPS cells from human urine cells may be a new noninvasive strategy for regenerative medicine in cardiomyocytes.

## RESULTS

### Generation of human iPS cells with pre-defined reprogramming factors

**Fig. 1.**
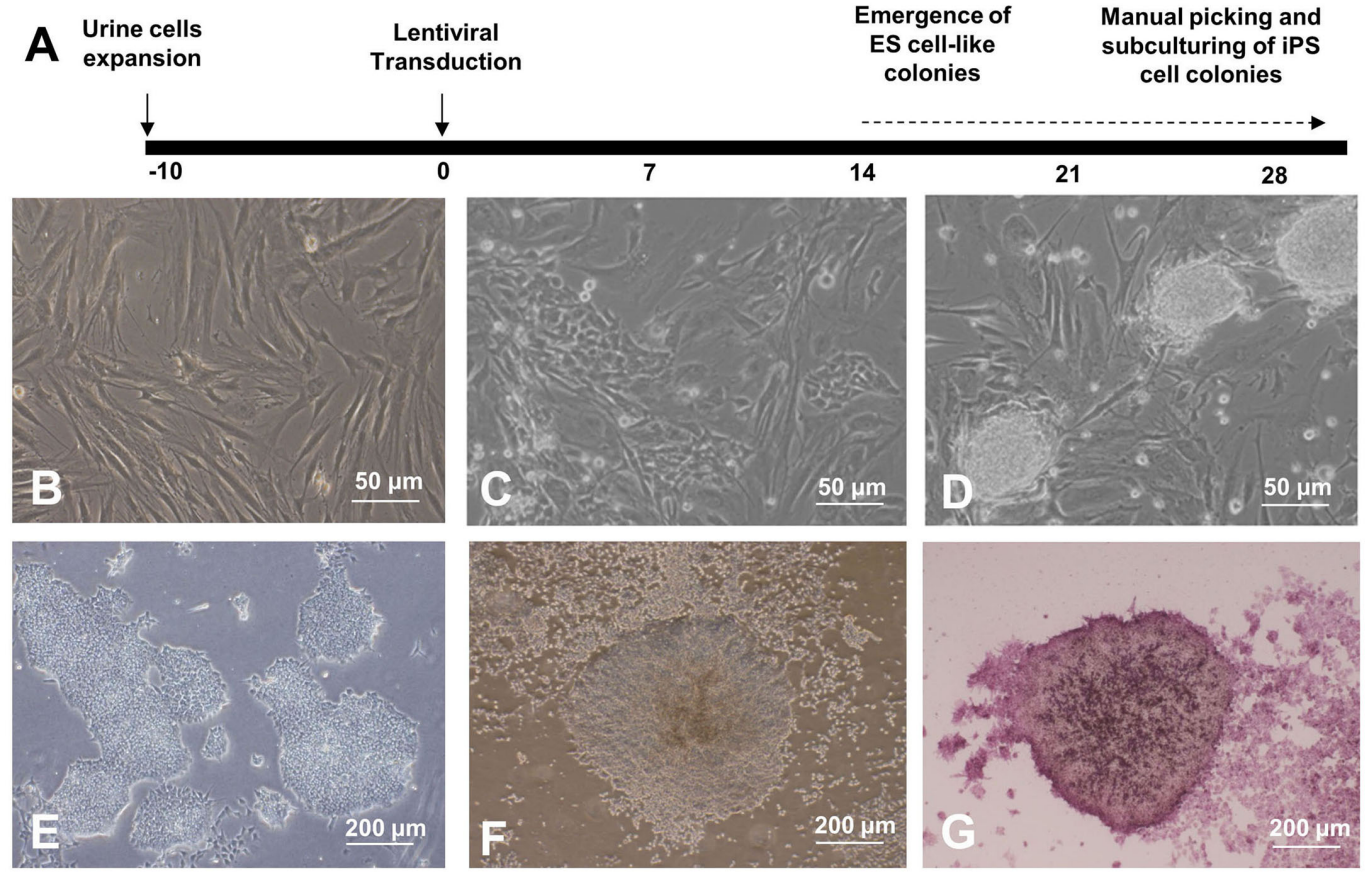
**Generation of induced pluripotent stem cells (iPSCs) from urine cells (UC).** (A) Schematic representation of iPSC generation from UC. SKOM refers to the four exogenous factors Sox-2, Klf-4, Oct-4, and c-Myc. Representative phase contrast photographs of UC of passages 2 to 4 at different points of the protocol. (B) UC of passages 2 to 4 before infection. (C) UC at day 7 after infection. (D) Emergence of ES cell-like colonies at day 14 after infection. (E) Hand-picked putative human iPSC colony subcultured to new MatrigelTM-coated wells at day 28 after infection. (F) Morphology of hand-picked putative human iPSC colony after subculture, and at 10 passages. (G) Intensive alkaline phosphatase expression in putative human iPSC colony (purple color). Scale bars in B-D represent 50 μm and in E-G represent 200 μm.

Human iPS cells were generated from 2-4 passage urine-derived epithelial cells and were obtained from two healthy Chinese adults using four reprogramming factors (*Oct4*, *Sox2*, *Klf4* and *c-Myc*). The study protocol was summarized in Fig. 1A. Urine-derived cells at passage 2 to 4 with a density of 1×10^5^ cells per well in a 6-well culture dish were ready for lentiviral transduction (Fig. 1B). A small amount of compact clusters (5-10 per well), in which cell morphology was similar to human embryonic stem cells (ESCs), with cobblestone appearance, prominent nucleoli, and distinct cell boundaries (Fig. 1), was observed after about 1 to 14 days of lentivirus transduction (Fig. 1C,D). In the mTeSR1TM medium (Fig. 1E), these putative human iPS cells were handpicked as subcultures and showed progressive increase in size with distinct alkaline phosphatase expression (Fig. 1F,G).

### Pluripotency markers

In order to test whether our putative human iPS cells express human embryonic stem cell markers, such as Oct4, Nanog, SSEA-4 and TRA-1-60, immunofluorescence staining was performed on well-defined colonies after 10 passages. Positive results were obtained from the staining of Oct4, SSEA-4, Nanog and TRA-1-60 (Fig. S1A), which was in consistent with flow cytometry analysis: Oct4 (99.2±6.0%), SSEA-4 (98.8±6.7%), Nanog (98.6±7.1%) and TRA-1-60 (99.6±6.6%) (*n*=3, Fig. S1B). These results indicated that the reprogrammed human urine-derived epithelial cells express these typical ESC markers.

### Pluripotency tests of putative human iPS cells *in vitro* and *in vivo*

**Fig. 2.**
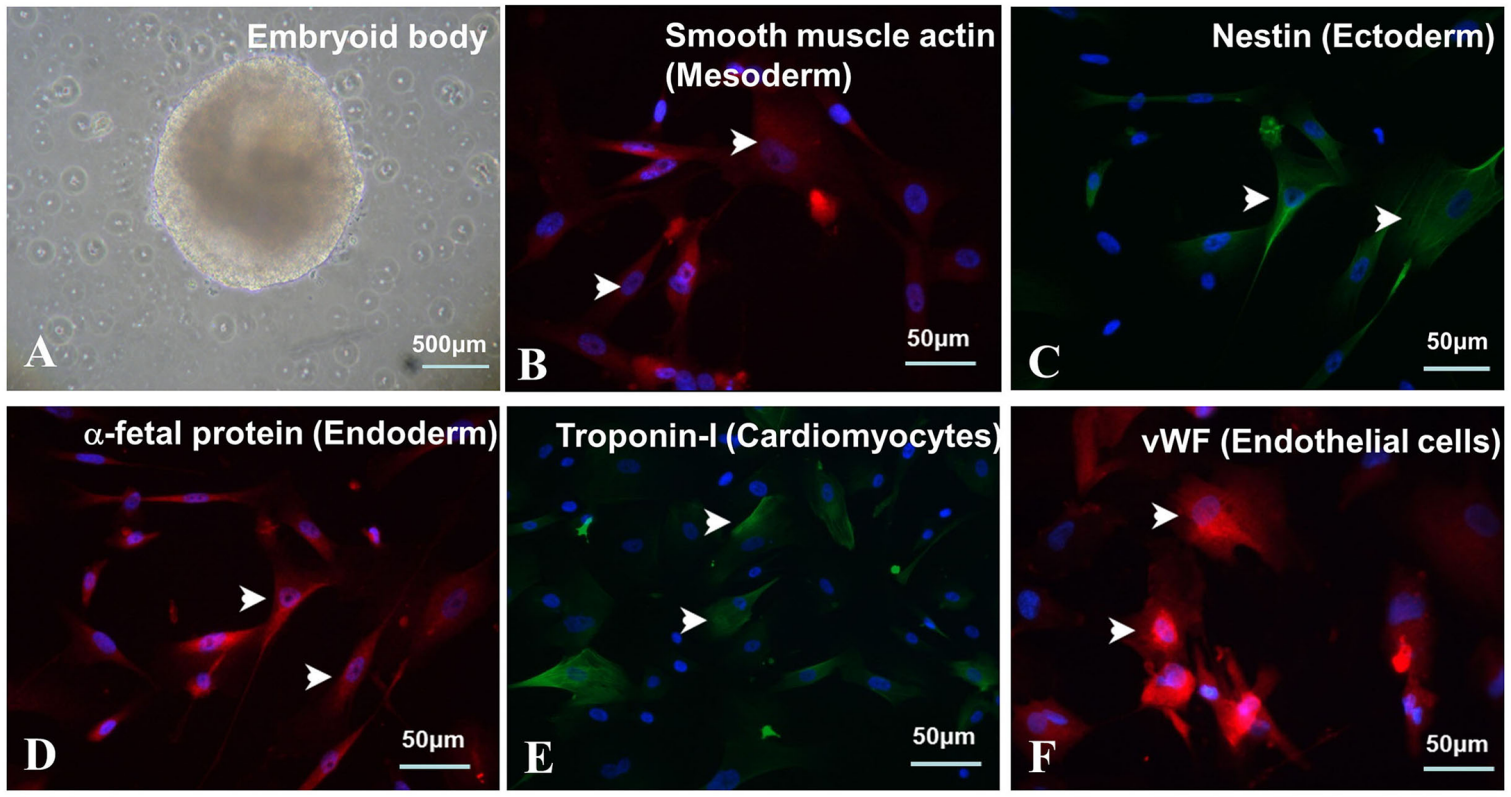
**Differentiation of putative human iPS cells generated from urine cells *in vitro*.** (A) Embryoid body formation in suspension culture with spontaneously differentiation into the three germ layers (arrowheads in B-F): (B) smooth muscle actin (mesoderm), (C) nestin (ectoderm) and (D) alpha-fetoprotein (endoderm). In addition, human iPSC spontaneously differentiated into cardiomyocytes (troponin-I, E) and endothelial cells (vWF, F). Scale bars in A represent 500 μm and in B-F represent 50 μm .

We conducted pluripotent tests of human iPS cell lines both *in vitro* and *in vivo*. These hypothetical human iPS cells were easily aggregated into spherical embryoid bodies as embryonic stem cells (Fig. 2A) after induction of embryonic body differentiation by hanging drop method. After germination, the cells grown spontaneously from the embryoid bodies expressed three primitive germline markers: endoderm, alpha-fetoprotein; ectoderm, nestin; and mesoderm, anti-smooth muscle actin (Fig. 2B-D). Moreover, Troponin-I-positive cell clusters indicating myocardial lineage and vWF-positive cells suggesting endothelial cell lineages (Fig. 2E,F) were also observed.

**Fig. 3.**
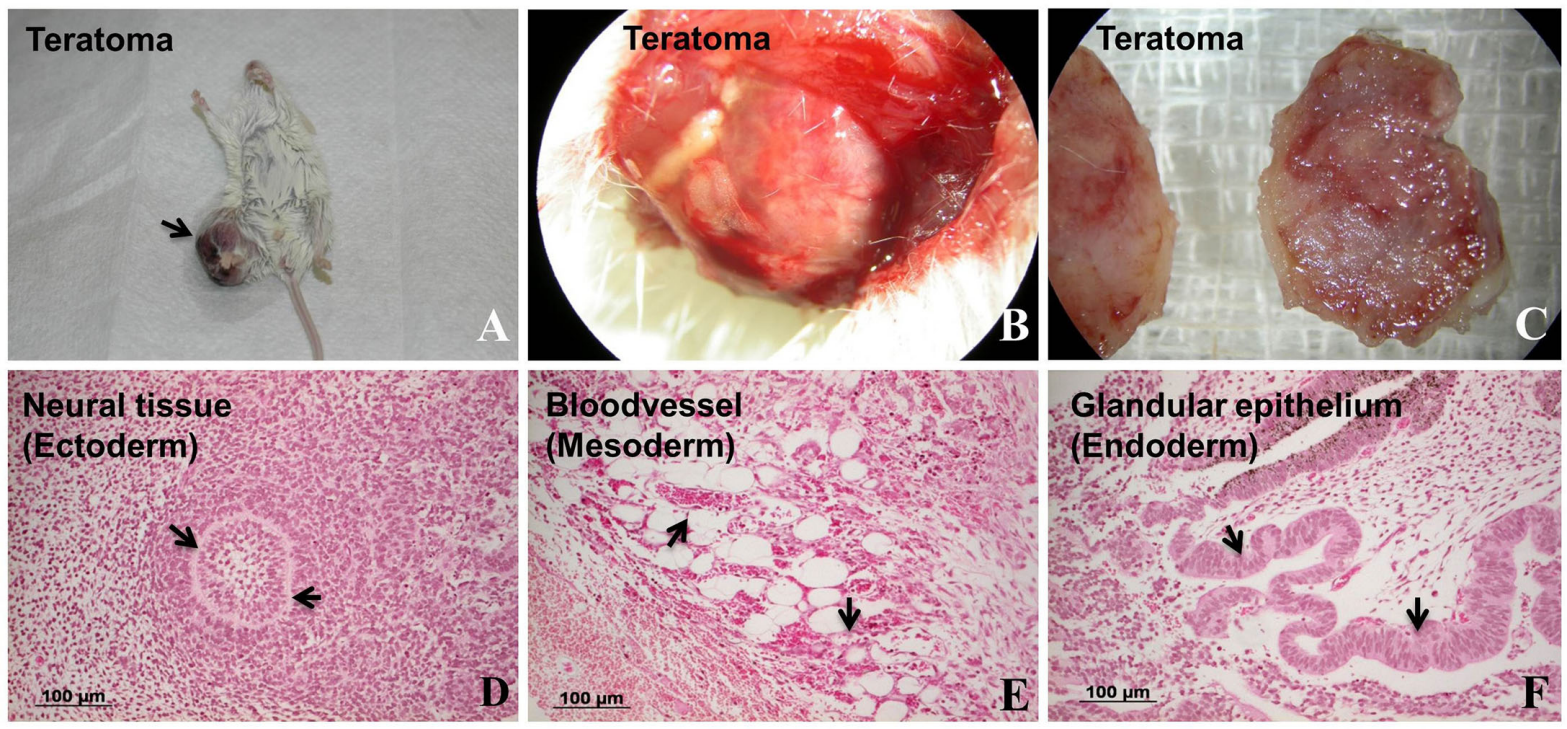
**Differentiation of putative human iPSCs generated from urine cells *in vivo*.** Human iPS cell-induced teratoma formation 5 weeks after subcutaneous injection in NOD/SCID mice. Staining with hematoxylin/eosin of the three germ layers (arrowheads) in the teratoma. The scale bars in D-F represent 100 μm.

We injected our putative human iPS cells subcutaneously into three nonobese diabetic/severe combined immunodeficiency (NOD/SCID) mice for teratoma-like mass formation. After 5 weeks from injection, well-encapsulated tumors showed differentiated tissues of the three embryonic germ layers in two of these three mice (Fig. 3A-F).

### Identification of cardiomyocytes derived from iPS cells

**Fig. 4.**
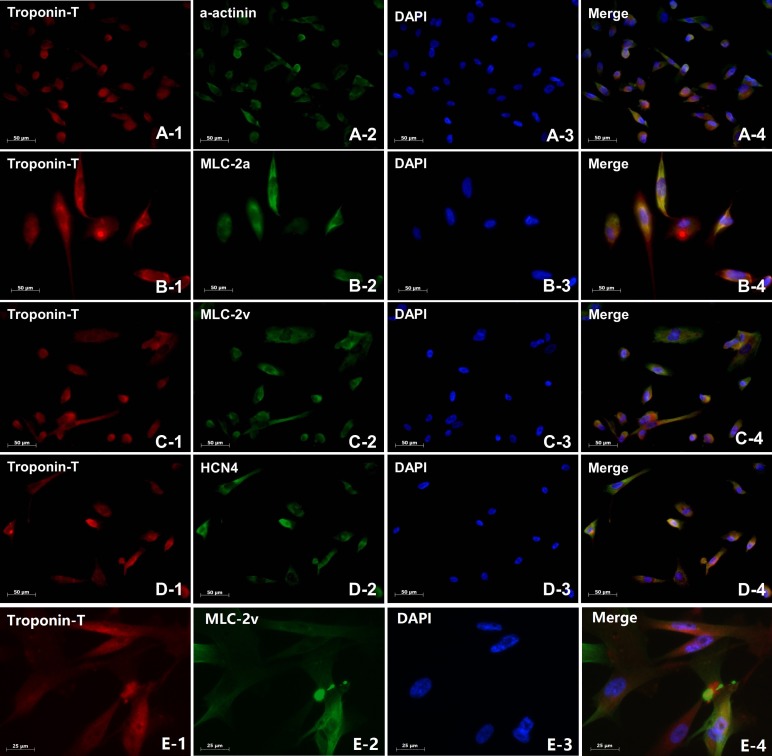
**Immunofluorescence staining of cardiomyocytes derived from iPS cells.** Immuno-labeling of single cells dissociated from a BC with antibodies against canonical pan-cardiac-specific marker-Troponin T with a-actinin (A1-4), atrial myocyte-specific MLC-2a (B1-4), ventricular myocyte-specific MLC-2v (C1-4) and pacemaker-specific HCN4 (D1-4) and higher magnification image for ventricular myocyte-specific MLC-2v (E1-4). The scale bar represents 50 μm (for A-D) and 25 μm (for E).

Spontaneous beating occured in the embryoid body (EB) at approximately 14 to 21 days, and the rate of beating was 29-60 beats per min (Movie 1). On day 21 after differentiation, the maximum percentage of beating EBs (50%) was reached. EBs were dissected by minimally invasive surgery and dissociated with collagenase II into single cell clusters, which yielded spontaneously beating single putative cardiomyocyte (Movie 2). Immunofluorescence assay showed canonical pan-cardiac-specific marker in beating cells, including troponin-T, alpha-actin (Fig. 4A), atrial myocyte-specific MLC-2a (Fig. 4B), ventricular myocyte-specific MLC-2v beating cells (Fig. 4C) and pacing cell-specific HCN4 (Fig. 4D). Higher magnification image for ventricular myocyte-specific MLC-2v was shown in Fig. 4E. To further confirm cardiac differentiation, flow cytometry was performed to determine the percentage of cardiomyocytes identified by troponin-T-positive cells. Experiments were technically repeated twice, with two duplicates from each donor. A total of 1×10^5^ cells stained with rabbit or mouse anti-human Troponin T (Millipore), MLC-2a (Synaptic), and MLC-2v antibodies (Synaptic), were analyzed. We obtained approximately 4×10^4^ cells with Troponin T positive. The percentage of cardiomyocytes was 40.5±2.5%, showing better efficiency compared to previous studies (Ivashchenko et al., 2013; Priori et al., 2013). Most troponin T-positive cardiomyocytes have ventricular phenotypes (62.5±3.5%), and the rest (45.8±3.0%) showed immunofluorescence-based atrial phenotypes (Fig. S2A). Positive percentages of markers for pluripotency of putative human iPS cells and cardiac-specific markers were shown in Fig. S2B,C. All of these positive markers in beating cells confirmed their cardiac identity.

### Action potential recorded from spontaneously beating clusters

Stable action potential (AP) recording was obtained from the 27 beating clusters (BCs) of the same batch of EBs. Traces were arranged after 21-60 days post differentiation. Although 16.7% (5/30) were classified as atrial-like APs and 83.3% (25/30) were ventricular-like (Fig. S3), the figure showed an almost continuous range of AP morphology, which directly lead to the subjective nature of recognition based on AP profiles. It was notable that our APs did not meet the criteria to be nodular-like [i.e. ratio (RO)≤1.5+low amplitude+smaller negative MDP+low Vmax]. The standard used to distinguish between atrial-like (RO≤1.5) action potentials and ventricular-like (RO>1.5) action potentials in this study was according to APD_30-40_/APD_70-80_ ratios (RO) (Ma et al., 2011).

## DISCUSSION

In present study, we described the generation of iPS cells from human urine-derived epithelial cells using four transcription factors (*Oct4*, *Sox2*, *Klf4* and *c-Myc*), without feeder cells or antibiotic selection. The cardiac markers, including Troponin-T, alpha-actin, MLC-2a, MLC-2v, and HCN4, were tested. The electrophysiological action potential was recorded in iPS cells beating clusters. These results show that mature functional cardiomyocytes with cardiac-specific markers and cardiomyocyte potential can be generated from urine-derived cells non-invasively via cellular reprogramming methodologies.

Of notable strength was that we derived cardiomyocytes following strict differentiation protocols using serum-free, chemically defined media in stag-specific manner containing Activin A, bFGF, BMP4, DKK-1 and VEGF as described previously (Kattman et al., 2006; Yang et al., 2008), which could significantly improve the efficiency of cardiomyocyte differentiation. Immunofluorescence analysis confirmed the differentiation of iPS cells into cardiomyocyte by observation of the cardiac-specific markers (Troponin-T, α-actinin, MLC-2v, MLC-2a and HCN4). The percentage of cardiomyocytes was 40.5±2.5%, which is higher than other methods (Ivashchenko et al., 2013; Priori et al., 2013). Similarly, the most cardiomyocytes with Troponin-T positive (62.5±3.5%, ventricular myocyte-specific MLC-2v) presented the preference of a ventricular phenotype, and the remainder indicated an atrial phenotype (atrial myocyte-specific MLC-2a) based on immunofluorescent staining according to the results of flow cytometry. Furthermore, spontaneously beating outgrowths appeared approximately 14 to 21 days in embryoid bodies with beating rates ranging from 29-60 beats/min after cardiac differentiation. All of these positive markers and spontaneously beating cells confirmed that the iPS cells had been differentiated into cardiomyocytes *in vitro*.

To further study cardiomyocytes generated from iPS cells, we used the microelectrode recording of spontaneous beating cells to systematically study their electrical properties after 21 to 60 days of EBs formation. Consistent with previous studies (Ivashchenko et al., 2013; Priori et al., 2013), our experiments identified two types of cardiac muscle cells that resembled the atria and ventricles. As expected, the salient feature of atrial cells (RO≤1.5) was the short duration of action potential and lack of plateau phase, whereas ventricular cells (RO>1.5) exhibited a typical plateau phase, and therefore had the longest action potential duration (APD) (Ma et al., 2011). What's more, various impalements from each BC group presented an action potential with a comparative morphology, demonstrating that each bunch comprised of one major cardiomyocyte sort. These outcomes are practically identical to those beforehand depicted in iPS-determined BCs (Ivashchenko et al., 2013; Priori et al., 2013).

Due to the small dimension of BCs, there were likely to be multiple phenotypes, but were masked as a result of electrical coupling properties (Kattman et al., 2006; Ma et al., 2011; Ivashchenko et al., 2013). Thus, these observations further supported that the iPS cells from urine cells had been differentiated into cardiomyocytes by functional evaluation.

Urine-derived cells reprogrammed by lentiviral transduction of pre-defined transcription factors is a demonstration of feasibility of generating human iPS cells to be differentiated into cardiomyocytes. However, genomic integration is an essential process for lentiviruses in reprogramming. *Klf4* and *c-Myc* are oncogenes, which can initiate tumor growth *in vivo*. We did not assess the levels of genomic disruption in this study. According to previous studies, the method of using hanging drop method for lentiviral transduction of iPS cells provides a suitable and sufficient strategy for the gene transfer, and its toxicity is less than the conventional suspension method (Zare, 2016). To avoid potential genomic disruption in the future, there will be a shift from using lentiviruses to other vectors without requirement of genomic integration, such as Sendai viral vectors (Lamb, 1996; Nishimura et al., 2011) and Epstein-Barr virus episomal vectors (Lindner and Sugden, 2007). Our study provides a platform for further investigating the possibility of iPS-derived cardiomyocyte generated from urine cells in cardiac cell therapy. The results may lead to a less expensive alternative, or at least a supplemental method, to generate cardiomyocytes in a totally noninvasive manner from an easily accessible source. By serving as models of cardiac diseases, it is likely to fundamentally alter the way in which cardiovascular disorders are studied and advanced therapies are developed.

## MATERIALS AND METHODS

### Ethical statement

This study was approved by the Institutional Review Board of the First Affiliated Hospital of Soochow University (Permit Number: 16-0712). The two volunteers involved in this study provided written informed consent. This study was conducted in strict accordance with the recommendations of the Guide for the Care and Use of Laboratory Animals of Chinese National Institutes of Health. The agreement was approved by the Animal Experimental Ethics Committee of the First Affiliated Hospital of Soochow University (Permit number: 16-1148). All operations on animals were performed under anesthesia of pentobarbital sodium and minimized suffering.

### Urine sample collection and urine-derived cells expansion

We collected 30 ml midstream urine, respectively, from two healthy Chinese volunteers (one male, 62 years old; another female, 58 years old) into sterile containers. The urine sample was transferred to a sterile 50 ml tube and centrifuged at 400 ***g*** for 10 min at room temperature. We then aspirated the supernatant carefully and left only 1 ml in the tube. The particles in the remaining 1 ml of urine were gently and individually suspended and then collected (if there were multiple urine tubes) into a single 50 ml tube. We then added 10 ml of washing buffer, and the sample was centrifuged at 200 ***g*** for 10 min at room temperature and the supernatant was discarded. 1 ml of primary medium was added to resuspend the cell pellet and the volume was transferred to a single well of a 12-well plate [precoated with 0.1% (wt/vol) gelatin]; 1 ml of primary medium was added to the cells at 37°C. The cells were incubated for 24 h.

After plating for approximately 96 h, all but 1 ml medium was aspirated, then 1 ml of proliferation medium was added. We renewed half of the proliferation medium every day and reserved the other half. When the urine cell culture was sufficiently dense and ready for passaging, all cells were split into a new well of 12-well plate for further expansion. This is considered to be passage 1. We continued to culture the cells and renew the medium every other day. If more than 100,000 cells were assessed by counting, they could be split into a well of six-well plate and prepared for infection.

### Lentivirus infection and human iPS cells generation

We adopted the following lentiviral plasmids: pSin-hSox2 and pSin-hOCT4 (Addgene); h*c-Myc*-pCMV-Sport6 and hKLF4-pCMV-Sport6 (Open Biosystem) and subcloned into pSin plasmid (Addgene). The multiplicity of infection was 10.

Urine-derived cells at passages 2 to 4 were seeded in 6-well dishes with a density of 1×10^5^ cells per well and two rounds of 100 μl of each virus with 6 μg/ml polybrene (Sigma) were transduced at 37°C. The virus supernatants contained cDNAs of human *Oct4*, *Sox2*, *Klf4* and *c-Myc*. Two rounds of infection (12 h each) were conducted as described previously (Giorgetti et al., 2009). We added polybrene (Sigma) to increase infection efficiency. After that, we changed the culture medium of the transduced cells to a urine-derived cells culture medium. Respectively, we monitored the infection efficiency, which was close to 100%, referenced by GFP expression vector transduction. On day 4, trypsin digestion was performed with cells transduced by reprogramming factors. Routinely, 50,000 cells using human ESC medium (Invitrogen) were seeded and renewed daily. On day 5, we changed the medium to human ESC medium with 1 mM valproic acid (Sigma) until day 12. Then we changed to mTesR1 medium (StemCell). After infection, we changed the medium daily. On day 16, the colonies which were sufficiently huge with a human ESC-like preference, could be picked mechanically and extended in human ESC medium on feeders or on mTesR1 medium on Matrigel.

### Flow cytometry of reprogrammed iPS cells

Reprogrammed iPS cells were collected separately by digestion with TripLE solution (Invitrogen). We used 2% paraformaldehyde containing 0.05% Triton X-100 to fix and obtain permeabilized cells. After that, we stained the iPS cells with rabbit or mouse anti-human Oct4, Nanog, Sox2, SSEA-4 and TRA-1-60 antibodies (Stemgent, San Diego, CA) for analysis on FC500 flow cytometry (Beckman, Fullerton, CA) using CXP analysis software (Beckman). We assumed rabbit or mouse isotypic antibodies as negative controls respectively.

### Alkaline phosphatase and immunofluorescence staining of iPS cells

We followed the pre-established protocol from the manufacturer (Stemgent) to perform alkaline phosphatase staining. We also did immunofluorescence staining with Oct4, Nanog, SSEA-4 and TRA-1-60 pluripotency markers (Stemgent). Positive colonies were observed at 30 min after addition of goat anti-rabbit IgG H+L Alexa 594 and rabbit anti-mouse IgG H+L Alexa 488 (Molecular Probes).

### *In vitro* differentiation of iPS cells

For embryoid body (EB) formation, after dissociating the putative human iPS colonies into individual cells by TripLE solution (Invitrogen), drops of 1000 cells were suspended in 20 μl culture medium containing no bFGF on petri dish lids for 48 h. The embryoid bodies were thereafter suspended in DMEM medium containing 10% FBS, 1 NEAA and 1 mM L-glutamine for 5 days, and then plated in a gelatin-coated 12-well plate in the same medium for another 7 days. The embryoid bodies were then settled and restained with a human embryonic germination characterization kit (Chemicon, Rolling Meadows, IL).

### Teratoma formation *in vivo*

After injecting putative human iPS cells subcutaneously into C17 NOD/SCID mice (1×10^6^ cells per site) for 5 to 8 weeks, we resected the tumors and fixed them in 4% buffered formalin. After that, we conducted paraffin sectioning. Histological analysis was performed using hematoxylin and eosin staining.

### Cardiomyocytes differentiation from iPS cells *in vitro*

The undifferentiated iPS cells were maintained in a serum-less, non-feeder condition and routinely expanded with mTeSR1 medium (Stem Cell Technologies) on coated dishes (BD Biosciences). We used a serum-free, chemically defined medium with activin A, bFGF, BMP4, DKK-1 and VEGF supplements using a directional differentiation protocol to obtain cardiomyocytes in the stage-specific manner described previously (Kattman et al., 2006; Yang et al., 2008). Our optimized protocol produced up to 90% contraction clusters of the total embryoid bodies at 8 to 10 days after differentiation. Twenty days after differentiation, the beating clusters were dissected by a glass knife and plated on gelatin coated dishes with EB10 medium (Gemini), 100 mM nonessential amino acids and 100 mM b-mercaptoethanol (Gibco) (Xue et al., 2005). A single cell dissociated from the contractile cluster with collagenase II (Worthington, Lakewood, NJ) was plated in a petri dish (Invitrogen) for confirmation of its cardiac characteristics.

### Flow cytometry of differentiated iPS cells

Individual cells were isolated from the contracticle cluster by digesting with the TripLE solution (Invitrogen). Then 2% formaldehyde with 0.05% Triton x-100 was used to fix and permeate cells. Cells were stained with rabbit or mouse anti-human Troponin T (Millipore), MLC-2a (Synaptic, Göttingen, Germany), and MLC-2v antibodies (Synaptic), and analyzed by CXP analysis software (Beckman Coulter). Rabbit or mouse isoforms were used as negative controls, respectively.

### Immunohistochemistry of differentiated iPS cells

We used trypsin to isolate each single cell and plated them in fibronectin-covered dishes for no less than 5 days before immunostaining. We then washed the cells and took PBS and 4% paraformaldehyde for settlement for 15 min. From that point, the settled cells were permeabilized with 0.1% Triton-x, blocked with 5% fetal calf serum and hatched overnight with essential antibodies, and after that brooded at room temperature for 1 h at a weakening of 1:1000. After the last washing, coverslips were mounted with Prolong Gold Antifade (Molecular Probes). The marked cell pictures were achieved by fluorescence laser examining magnifying instrument (Zeiss). In our study, the following primary antibodies used were used: anti-Troponin T (Millipore, 1:300 dilution), a-actinin (Sigma, 1:200 dilution), MLC-2a (Synaptic, 1:200 dilution), MLC-2v (Synaptic, 1:200 dilution) and HCN4 (Chemicon, 1:50 dilution). The second antibody was donkey anti-mouse IgG Alexa488, Alexa594 and donkey anti-rabbit IgG Alexa488 (Invitrogen).

### Action potential recording of differentiated iPS cells

The action potential was recorded at 37±0.5°C from the spontaneously beating cluster under HEPES-Tyrode's solution with following ingredients (mM): 140 NaCl, 4 KCl, 1 MgCl_2_, 10 HEPES, 10 D-glucose and 2 CaCl_2_ using a sharp microelectrode (40–60 mV filled with 2.7 M KCl) referenced to the ground; pH was adjusted by NaOH (1N) to 7.4. The microelectrodes were connected to the amplifier (Axopatch, Foster City, CA) and the pClamp9.2 software (Axon, Union City, CA) operated in bridged mode. The GFP fluorescence was observed using a xenon arc lamp at 488/530 nm (excitation/emission). The contraction of beating cells was captured using the video edge detection system (Crescent, Sandy, UT) and the FTM800NH/HGI camera (Philips) at 60 Hz refreshing rate. All signals were digitized and stored on magnetic media and analyzed by Spike 2 (Cambridge Electronics Design, Cambridge, UK).

## Supplementary Material

Supplementary information
