# Supplementary material for: In vitro and in vivo differentiation of induced pluripotent stem cells generated from urine-derived cells into cardiomyocytes
Source: Biol Open. 2017 Dec 6;7(1):bio029157. doi: 10.1242/bio.029157 (PMC5829497; doi:10.1242/bio.029157)
Supplement: Supplementary information [file biolopen-7-029157-s1.pdf]

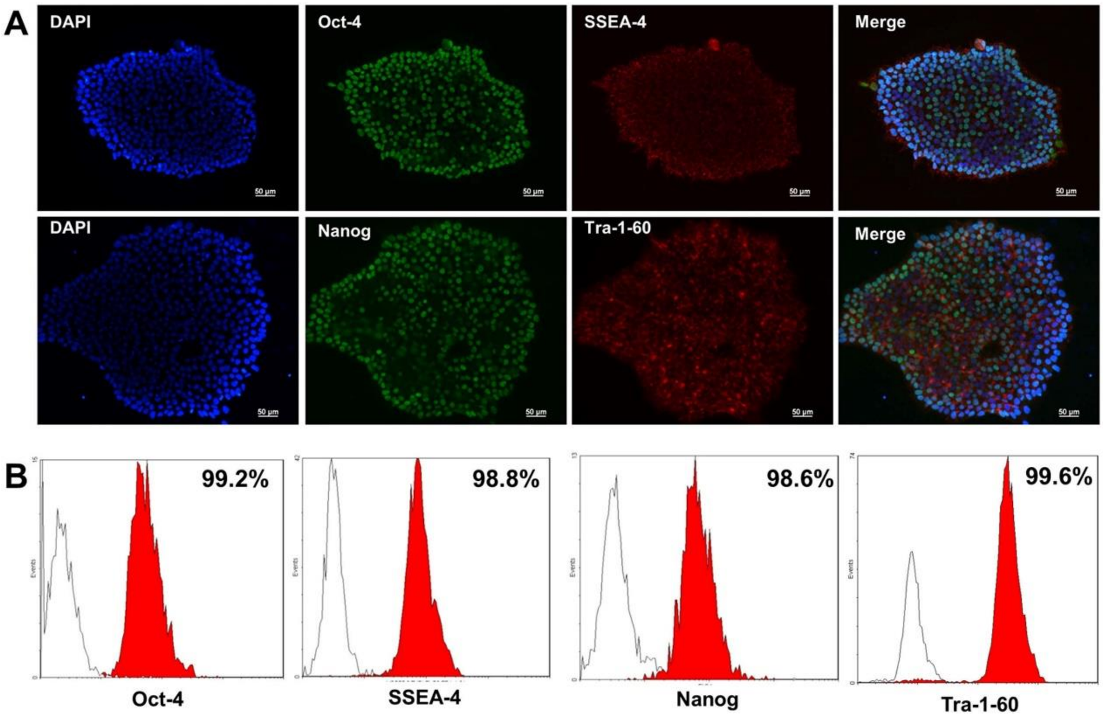

Fig. S1 Characterization of the putative human iPS cells.

(A) Immunofluorescence analysis of putative human iPS cells. The colonies expressed ESC pluripotency markers including Oct-4, Nanog, SSEA-4 and TRA-1-60. (B) Flow cytometry analysis of putative human iPS cell lines for pluripotency markers: Oct-4, SSEA-4, TRA-1-60, and Nanog. The scale bar represents 50 µm.

Biology Open • Supplementary information

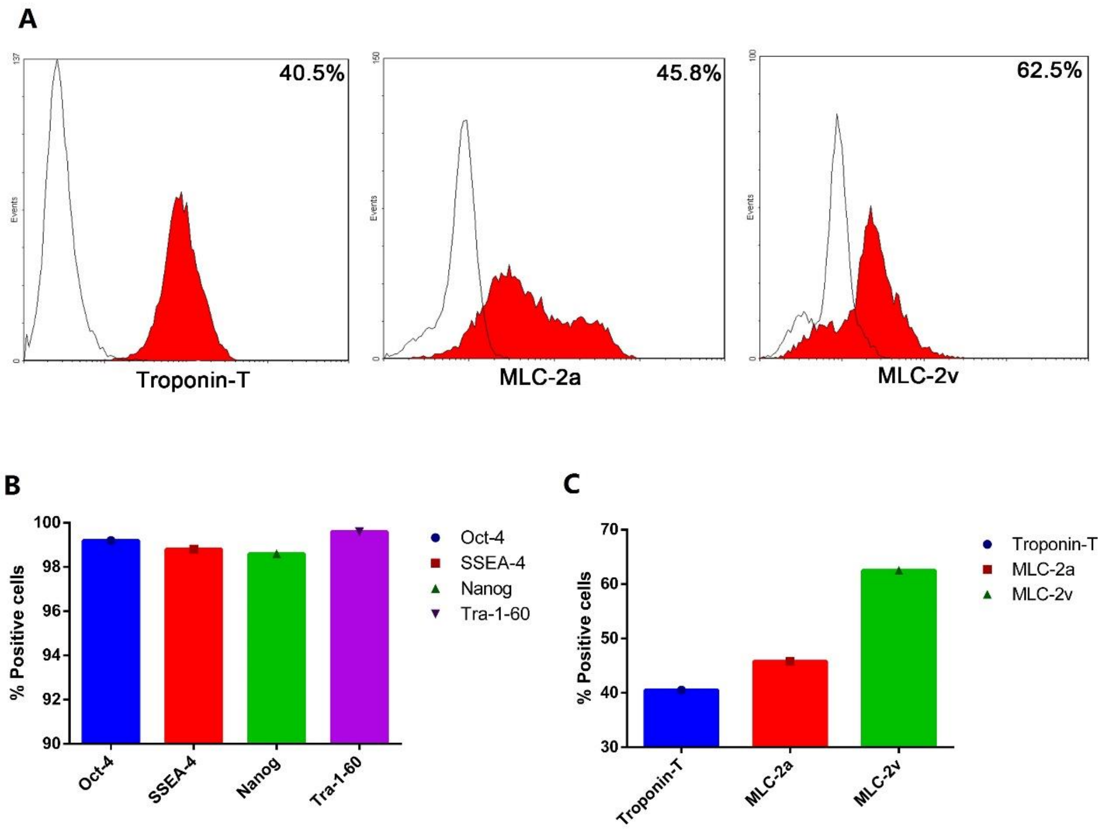

Fig. S2 Efficiency of cardiac differentiation from iPS cells.

(A) Flow cytometry analysis of cardiac differentiation from iPS cells for cardiac specific markers: Troponin-T, MLC-2a, and MLC-2v. (B) Positive percentages of markers for pluripotency of putative human iPS cells. (C) Positive percentages of cardiac specific markers.

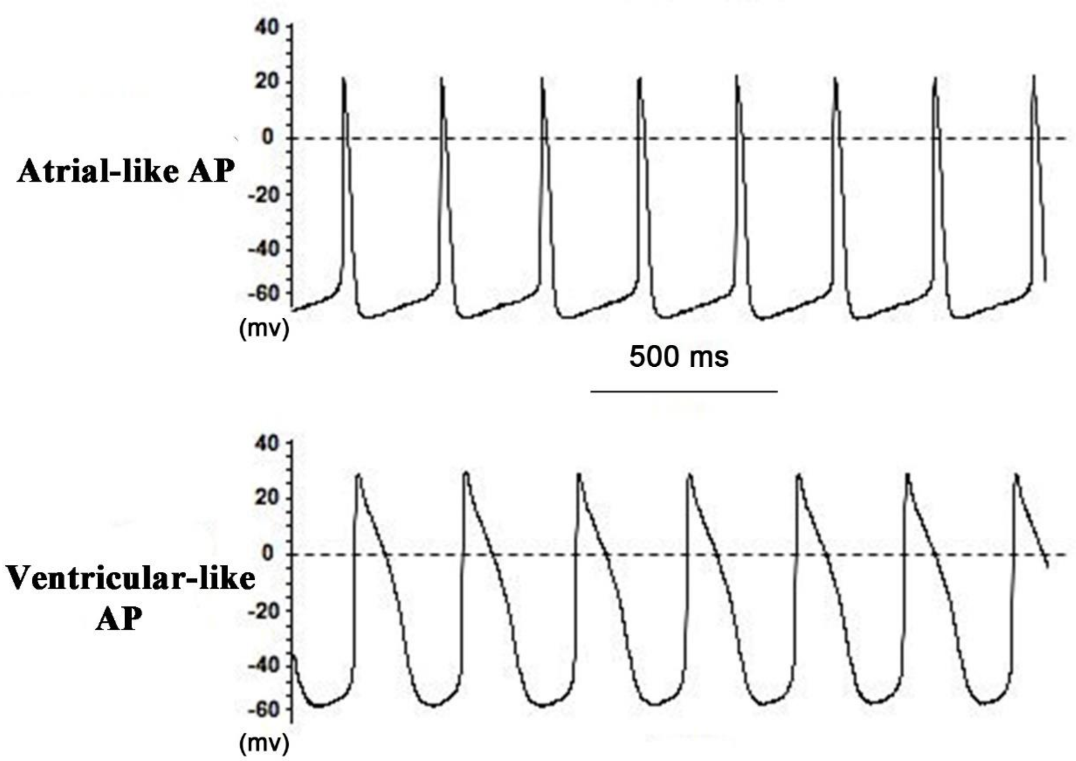

Fig. S3 AP recordings obtained from 30 BC studied from the same batch of EBs.

Atrial-like APs (16.7%); all others were classified as Ventricular-like (83.3%).

Biology Open • Supplementary information

Movies

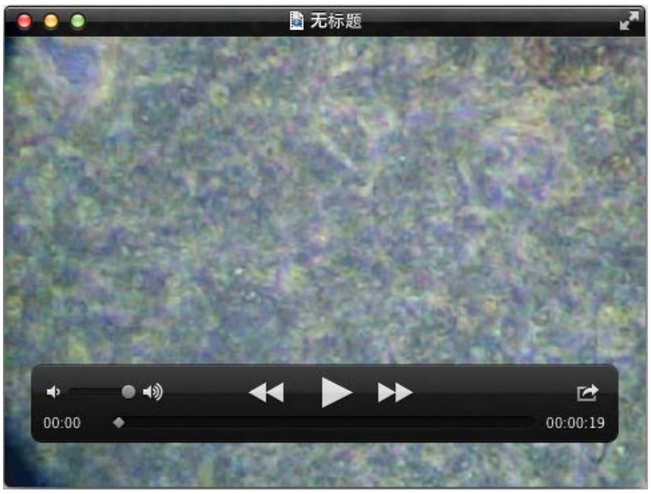

Movie 1 Spontaneously beating outgrowths in embryoid bodies.

Spontaneous beating occured in the embryoid body at approximately 14 to 21 days. Captured by FTM800NH / HGI camera (Philips).

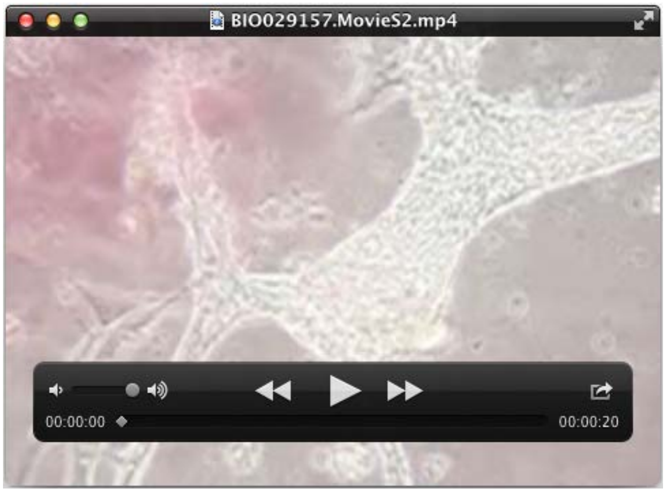

Movie 2 Spontaneously beating single putative cardiomyocyte.

Spontaneously beating single putative cardiomyocyte. Captured by FTM800NH / HGI camera (Philips).
